# Hepatoprotective Effect of Quercetin on Endoplasmic Reticulum Stress and Inflammation after Intense Exercise in Mice through Phosphoinositide 3-Kinase and Nuclear Factor-Kappa B

**DOI:** 10.1155/2016/8696587

**Published:** 2016-07-18

**Authors:** Yuhan Tang, Juan Li, Chao Gao, Yanyan Xu, Yanyan Li, Xiao Yu, Jing Wang, Liegang Liu, Ping Yao

**Affiliations:** ^1^Department of Nutrition and Food Hygiene, Hubei Key Laboratory of Food Nutrition and Safety, Ministry of Education Key Laboratory of Environment and Health and MOE Key Lab of Environment and Health, Key Laboratory of Environment and Health (Wuhan), Ministry of Environmental Protection, and State Key Laboratory of Environment Health (Incubation), School of Public Health, Tongji Medical College, Huazhong University of Science and Technology, Wuhan 430030, China; ^2^National Institute for Nutrition and Food Safety, Chinese Center for Disease Control and Prevention, Beijing 102206, China

## Abstract

The mechanisms underlying intense exercise-induced liver damage and its potential treatments remain unclear. We explored the hepatoprotection and mechanisms of quercetin, a naturally occurring flavonoid, in strenuous exercise-derived endoplasmic reticulum stress (ERS) and inflammation. Intense exercise (28 m/min at a 5° slope for 90 min) resulted in the leakage of aminotransferases in the BALB/C mice. The hepatic ultrastructural malformations and oxidative stress levels were attenuated by quercetin (100 mg/kg·bw). Intense exercise and thapsigargin- (Tg-) induced ERS (glucose-regulated protein 78, GRP78) and inflammatory cytokines levels (IL-6 and TNF-*α*) were decreased with quercetin. Furthermore, quercetin resulted in phosphoinositide 3-kinase (PI3K) induction, Ca^2+^ restoration, and blockade of the activities of Jun N-terminal kinase (JNK), activating transcription factor 6 (ATF6) and especially NF-*κ*B (p65 and p50 nuclear translocation). A PI3K inhibitor abrogated the protection of quercetin on ERS and inflammation of mouse hepatocytes. SP600125 (JNK inhibitor), AEBSF (ATF6 inhibitor), and especially PDTC (NF-*κ*B inhibitor) enhanced the quercetin-induced protection against Tg stimulation. Collectively, intense exercise-induced ERS and inflammation were attenuated by quercetin. PI3K/Akt activation and JNK, ATF6, and especially NF-*κ*B suppression were involved in the protection. Our results highlight a novel preventive strategy for treating ERS and inflammation-mediated liver damage induced by intense exercise using natural phytochemicals.

## 1. Introduction

Moderate exercise provides benefits to health, weight, and mood management. Accumulating evidence has demonstrated that regular physical activity enhances the antioxidant and anti-inflammatory properties and functional capacities of various tissues and organs. These factors minimize or prevent chronic problems, including the common nonalcoholic fatty liver disease [1]. Paradoxically, strenuous, prolonged, or intense exercises have been shown to be strong stressors with negative consequences, indicating a “J- or U-shaped association” between health and exercise; thus, there remains an incomplete understanding of the dose-response relationship between exercise and health [2]. Intolerable overexercise has been frequently associated with the occurrence of overtraining syndrome, which manifests as fatigue, impaired immune functions, repetitive tissue trauma, inflammation of muscles and viscera, and chronic health issues [3]. Injury and dysfunction of the liver, a vital organ responsible for the metabolism of nutrients and biochemical, inflammatory mediators and the detoxification of toxic substances, were first reported by Fojt et al. [4]. Subsequently, inflammation, oxidative stress, and disturbed lipid metabolism in animals and human models following vigorous overexercise have been reported in other studies [5–7]. In contrast with the widely studied exercise-induced heart injury models [8], studies of liver damage have drawn little attention due to the lack of evident clinical symptoms in early stages. A lack of understanding of liver disorders may impede the management of athlete health. Additionally, to prevent progression to advanced and fatal liver damage and thus to develop early intervention strategies, it is imperative to explore the molecular mechanisms underlying liver damage especially on endoplasmic reticulum stress (ERS) and inflammation induced by overexercise.

Recently, ERS has attracted particular interest due to its role in inducing inflammatory responses under various pathological conditions. Of note, moderate treadmill running has been found to attenuate skeletal ERS and improve insulin sensitivity in rats with insulin resistance [9]. Furthermore, moderate exercise has been shown to repress neuronal ERS and inflammation in aged presenilin 2 mutant mice [10]. Deldicque et al. [11] have found that endurance training promotes the unfolded protein response but attenuates inflammation in the soleus and tibialis anterior muscles and in the livers and pancreases of rats fed high-fat diets, suggesting a positive protective adaptation against further cellular stress. However, few studies have addressed the role of ERS in exhaustive exercise-related disorders, especially liver damage. Kim and his colleagues have observed evident ERS, oxidative stress, and inflammation in biopsies of vastus lateralis muscles after 200 km runs [12], further implying a link between ERS and overexercise. However, nuclear factor-kappa B (NF-*κ*B), Jun N-terminal kinase (JNK), and activating transcription factor 6 (ATF6) are considered crucial signaling pathways for ERS-initiated inflammation [9, 13]. Intense exercise has been reported to induce NF-*κ*B phosphorylation in human skeletal muscle [14] and ATF6 expression upregulation in the mouse hypothalamus, hippocampus, and cortex [15]. However, it has also been shown to inhibit JNK phosphorylation in the rat adrenal medulla [16]. The correlation between ERS and inflammatory damage and potential signaling pathways involved during vigorous exercise remain unclear.

Quercetin is the most common and widely distributed flavonoid in the plant kingdom, and it is especially abundant in apples (ranging from 2.1 to 7.2 mg/100 g) and onions (ranging from 28.4 to 48.6 mg/100 g). According to epidemical survey, the daily media intake of quercetin with a typical Western diet and in China was estimated to 10 mg and 5.96 mg/day, respectively [17]. In order to maximally exert its biological effect, quercetin should be assumed as purified dietary supplement but not natural food source. Quercetin has been found to have applicative uses in the military, athletic, and elderly populations and has also been considered in the preventive and therapeutic treatments of conditions and diseases related to oxidative and inflammatory damage. A systematic review [18] that pooled 11 studies totaling 254 human subjects has confirmed that quercetin provides significant benefits to human endurance exercise capacity and performance, despite variations in experimental designs [19]. Emerging evidence from our and other groups has shown that quercetin exerts hepatoprotective effects due to its antioxidant capacity, anti-inflammatory activity, and gene regulatory properties [19–21]. Furthermore, it has been suggested to alleviate inflammation by suppressing NF-*κ*B and, to some extent, JNK pathways [22, 23]. Moreover, Liu et al. [21] have found that quercetin prevents Pb-induced ERS at least in part due to its modulation of phosphoinositide 3-kinase (PI3K)/protein kinase B (Akt) and inositol-requiring enzyme 1 (IRE1)/JNK signaling pathways. However, the mechanistic role of the PI3K/Akt signaling pathway in the quercetin-mediated protection against intense exercise-induced ERS remains unclear. Furthermore, the mechanism responsible for transducing ERS signals into inflammatory injury has yet to be identified.

We designed the current study to determine the potential hepatoprotective effect of quercetin against intense exercise-induced hepatic ERS and inflammation damage. Furthermore, we characterized the underlying mechanisms involved in the regulation of the PI3K/Akt and ERS transducers (i.e., NF-*κ*B, JNK, and ATF6) during overexercise and in response to intervention by phytochemicals.

## 2. Materials and Methods

### 2.1. Chemicals and Materials

Quercetin (≥98%, HPLC), IV collagenase, thapsigargin (Tg), pyrrolidine dithiocarbamate (PDTC), SP600125, 4-(2-aminoethyl)benzenesulfonyl fluoride hydrochloride (AEBSF), and LY294002 were obtained from Sigma-Aldrich (Saint Louis, Missouri, USA). Assay kits for aspartate/alanine transaminase (AST/ALT) were purchased from Mindray (Shenzhen, China). Anti-glucose-regulated protein 78 (GRP78) rat monoclonal antibody was obtained from Santa Cruz Biotechnology (Santa Cruz, California, USA). Rabbit monoclonal IL, interleukin-6 (IL-6), PI3K (p85), Akt and phospho-Akt (Ser473) antibodies, anti-JNK rabbit monoclonal antibody, anti-p-JNK mouse monoclonal antibody, anti-TATA binding protein (TBP) rabbit polyclonal antibody, horseradish peroxidase- (HRP-) conjugated goat anti-rat IgG (secondary antibody), anti-HRP-conjugated goat anti-rabbit IgG, and anti-HRP-conjugated horse anti-mouse IgG were acquired from Cell Signaling Technology (Danfoss, Massachusetts, USA). Anti-glyceraldehyde-3-phosphate dehydrogenase (GAPDH) rabbit polyclonal antibody was obtained from Epitomics (Burlingame, California, USA). Anti-IRE1*α* rabbit polyclonal antibody, anti-p-IRE1*α* rabbit polyclonal antibody, anti-p-protein kinase RNA-activated-like ER kinase (PERK) rabbit monoclonal antibody, and anti-tumor necrosis factor (TNF-*α*) mouse monoclonal antibody were purchased from Abcam (Cambridge, British). Anti-p65 rabbit monoclonal antibody and anti-p50 rabbit polyclonal antibody were obtained from Cell Signaling and Protein Tech (Chicago, Illinois, USA), respectively. Western blot detection reagents (ECL) and reblot buffer were provided by Chemicon (Temecula, CA, USA). IL-6 and TNF-*α* ELISA kits are acquired from R&D Systems and Genzyme, respectively. Microsomal Ca^2+^ concentration determination and nuclear protein extraction kits were purchased from Genemed and Thermo, respectively. An ATF6 activity determination ELISA kit was obtained from Uscn Life Science. Other chemicals and organic solvents were of analytical grade and were purchased from local reagent retailers.

### 2.2. Animal Treatment and Exercise Protocol

Forty male adult BALB/C mice, obtained from Sino-British Sippr/BK (Shanghai, China) and with body weights of 18–20 g, were randomly divided into the following four groups containing ten animals each: (1) rested control (Ct); (2) intense exercise (Ex); (3) intense exercise plus quercetin supplementation (Ex + Qu); and (4) rested plus dietary quercetin supplementation (Qu) groups. Based on our preliminary test, quercetin was administered every day throughout the experiment (5 weeks) by gavage at a dose of 100 mg/kg·bw (10 mL/kg·bw), dissolved in physiological saline. The animals were subjected to an intense exercise program on the fourth week according to Marra et al. with some modifications [24]. The mice were forced to run on a motor-driven treadmill (Anhui, China) once a day for 7 successive days followed by a two-day rest period. They were acclimated to the treadmill exercise before training for two consecutive days. The exercise intensity (running speed) was set to a speed of 10 m/min for 10 min/day for the two acclimation days, after which time the animals were subjected to daily sessions of intense exercise for 28 m/min for 90 min/day following a 10 min warm-up period.

The animals were cared for according to the Guiding Principles of the Care and Use of Laboratory Animals published by the US National Institutes of Health. Animal experiments described in this study were approved by the Institutional Animal Care and Use Committee at Tongji Medical College, HUST (IACUC number: S407). The mice were housed in a temperature-regulated room (20–25°C) at a relative humidity level (60–75%) with a 12-hour light/dark cycle and provided with food and water ad libitum. The health statuses of the mice were monitored throughout the experiment by measuring the body weights on a weekly basis, in addition to monitoring food consumption, physical activity, and other measurable signs (general appearance, hair, eyes, nose, feces, urine, etc.) daily. The mice were immediately euthanized using a sodium pentobarbital solution (40 mg/kg·bw, 1% sodium pentobarbital dissolved in physiological saline, 4 mL/1 kg·bw, ip) after the final exercise session. Serum samples were prepared from blood by centrifugation at 3500 g for 10 min at 4°C (Eppendorf 5810R, Hamburg, Germany). Fresh liver samples were fixed for histopathological examination, and flash-frozen samples were used for Western blot and enzymatic analyses.

### 2.3. Determination of Serum Aminotransferase and Cytokine Levels and Hepatic Oxidative Stress Status

The serum levels of aminotransferases (AST and ALT) and inflammatory markers (TNF-*α* and IL-6) were measured by enzymatic kinetic methods and enzyme-linked immunosorbent assays according to the manufacturer's instructions with the relevant kits, respectively.

Malondialdehyde (MDA), ROS, reduced glutathione (GSH), and superoxide dismutase (SOD) activities were determined as previously described [25, 26]. Liver homogenate with isotonic saline (10%) was centrifuged at 3500 g for 10 min (4°C) to obtain the supernatant. The hepatic ROS assay was based on the oxidation of dihydroethidium (DHE), yielding a red fluorescent product, which was observed with a Nikon 2000S fluorescence microscope (Melville, NY) and analyzed by Image-Pro Plus software. The specific activities of the various enzymes and the MDA concentration in the liver were normalized to the total protein concentration and expressed as nmol/mg protein.

### 2.4. Liver Ultrastructural Examination

Exercise-induced ultrastructural changes were observed by conventional methods. Briefly, fresh liver fragments (1 mm cubes) were carefully collected, rapidly fixed in 2.5% glutaraldehyde (in phosphate buffer, pH 7.4), and then postfixed in 1% osmium tetroxide. Subsequently, the samples were dehydrated by graded alcohol and embedded in Epon 812 resin. Ultrathin sections were prepared using an Ultramicrotome (Leica UCT, Germany). The sections were stained with uranyl acetate and lead citrate and were subsequently visualized by transmission electron microscopy (FEI Tecnai G2 12, Netherlands). Digital images were captured with an integrated CCD camera.

### 2.5. Microsomal Extraction and Ca^2+^ Concentration Determination

Microsomal samples were extracted from the liver homogenate (0.1 M potassium phosphate buffer) by differential ultraspeed centrifugation (3500 g for 10 min, 10,800 g for 15 min, and 105,000 g for 60 min; Beckman, Optima L-90K, USA) at 4°C, according to previously reported methods [25]. Ca^2+^ concentrations were measured by fluorescence spectrophotometry using Fluo-4-AM.

### 2.6. Isolation, Culture, and Treatment of Mouse Hepatocytes

Mouse hepatocytes were isolated by a two-step collagenase perfusion technique from the abdominal aorta. The perfusions were purified via a Percoll gradient, as described by Cao et al. [27]. Freshly harvested hepatocytes were cultured on rat-tail collagen-coated plates and maintained in high glucose DMEM supplemented with glutamine, 10% fetal bovine serum, 32 IE/L insulin, 15 mM HEPES, 0.1 *μ*M hydrocortisone, 100 U/mL penicillin, and 100 U/mL streptomycin. The next day, the media were exchanged and supplemented with Tg (1 *μ*M), PDTC (300 *μ*M), SP600125 (20 *μ*M), AEBSF (300 *μ*M), LY294002 (30 nM), and quercetin (100 *μ*M) for 24 hours. Then, the cells and supernatants were collected for bioassays performed according to the corresponding experimental protocols. Cell viability was 80–95% (as determined by the trypan blue exclusion test) under these conditions.

### 2.7. Immunohistochemistry

Liver tissue samples fixed in 4% paraformaldehyde/PBS were embedded in paraffin and cut into 5 *μ*m thick sections. The tissue sections used to determine GRP78 expression were incubated with the appropriate primary antibody (diluted 1 : 100) overnight at 4°C. The immunostaining was visualized using 3,3′-diaminobenzidine following a 1-hour reaction with an HRP-conjugated-IgG antibody (diluted 1 : 200).

### 2.8. Real-Time Quantitative PCR Analysis

Total RNA was extracted from mouse liver tissues using TRIzol® reagent (Invitrogen, Carlsbad, CA) according to the manufacturer's instructions. The messenger RNA (mRNA) levels of the target genes were quantified by qRT-PCR using a SYBR green-based kit (TaKaRa BIO Inc., Dalian) with specific primers and an RT-PCR machine (7900HT, Applied Biosystems, Forster, CA). The mRNA level of *β*-actin served as an endogenous control and the results were calculated by the comparative 2^−ΔΔCt^ method. The forward and reverse primers for IL-6 (NM_031168.1) were GGG ACT GAT GCT GGT GAC AA and ACA GGT CTG TTG GGA GTG GT, respectively. In addition, the primers for TNF-*α* (NM_013693.3) were ATG GCC TCC CTC TCA GT and TTT GCT ACG TGG GCT AC, and those for *β*-actin (NM_007393.3) were TTC GTT GCC GGT CCA CAC CC and GCT TTG CAC ATG CCG GAG CC.

### 2.9. Western Blot Analysis

Mouse hepatocytes and liver tissues were lysed in RIPA Lysis Buffer (1% Triton X-100, 1% deoxycholate, and 0.1% SDS) or using a cell plasma and nuclear protein extraction kit according to the manufacturer's instructions. Tissue or cellular lysates with equal amounts of proteins mixed (3 : 1) with loading buffer were subjected to electrophoresis (Bio-Rad, USA) in acrylamide-SDS gels and subsequent electroblotting to polyvinylidene fluoride membranes (Millipore, Massachusetts, USA). The target proteins were probed with specific primary antibodies. Then, the blot membranes were incubated with species-specific HRP-conjugated secondary antibodies. The chemiluminescence intensities of the bands were subsequently detected using an ECL Plus Kit with a Western Blotting Detection System (Amersham Biosciences, Little Chalfont, UK). The optical densities of the bands were quantified by Gel Pro 3.0 software (Biometra, Goettingen, Germany). To eliminate background noise, the data were standardized to GAPDH as optical density values (OD/mm^2^).

### 2.10. Statistical Analysis

The data were expressed as the mean ± standard deviation (SD) and were analyzed using SPSS 12.0 software package by one-way analysis of variance (ANOVA). A probability value of 0.05 (two-tailed) was considered significant.

## 3. Results

All of the mice survived for the entire experimental period until they were sacrificed. There were no significant differences in the initial body weights among the 4 experimental groups. However, the weight loss of the overexercise-challenged mice was greater than that of nonoverexercised mice. Additionally, quercetin supplementation did not have an effect on weight change in the overexercise-exposed mice (Supplementary Figure  1; see Supplementary Material available online at http://dx.doi.org/10.1155/2016/8696587).

### 3.1. Quercetin Decreased Aminotransferase Release and Oxidative Stress and Alleviated Hepatic Ultrastructural Abnormalities in Overexercised Mice

**Figure 1:**
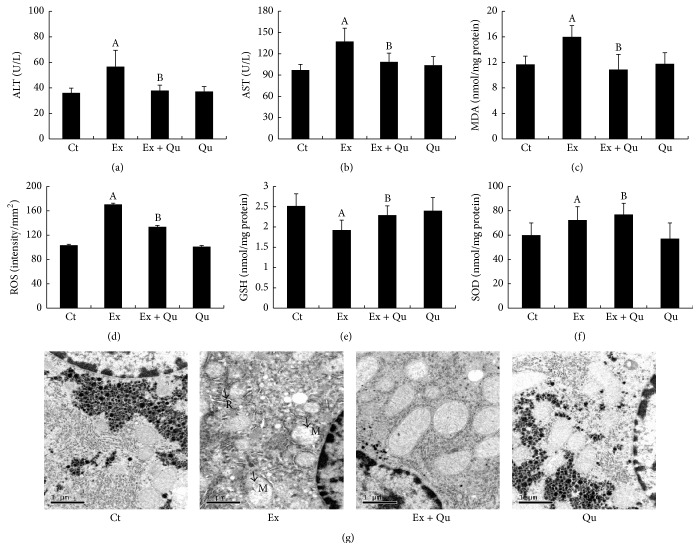
Effects of intense exercise exposure and quercetin pretreatment on serum aminotransferase levels, oxidative stress, and liver ultrastructural changes. Serum ALT (a) and AST (b) levels were measured using standard commercial assay kits. Quantification of MDA (c), ROS (d), GSH (e), and SOD (f) levels in liver tissues; the bars represent the mean ± SD (*N* = 3 for ROS determination, *N* = 10 for others). (g) Hepatic ultrastructures were observed by transmission electron microscopy. Significant differences (*P* < 0.05) are indicated by the different letters. A: versus rested control (Ct); B: versus intense exercise (Ex); Ex + Qu: intense exercise plus quercetin (100 mg/kg·bw); Qu: quercetin.

Intense exercise resulted in 1.6-fold and 1.4-fold increases in the serum ALT and AST levels, respectively, in the mice compared with those in the normal controls. The amounts of serum ALT and AST released from the intense exercise-exposed mice were decreased by 35% and 25%, respectively, as a result of daily quercetin pretreatment (Figures 1(a) and 1(b)). The MDA and ROS levels were increased in the intense exercise-challenged mice compared with the control mice (Figures 1(c) and 1(d)). Notably, tripeptide GSH was significantly consumed and depleted during intense exercise (Figure 1(e)), whereas a substantial increase in SOD activity was observed (Figure 1(f)). The pretreatment with quercetin reduced the accumulation of MDA and ROS and recovered the generation of GSH disturbed by rigorous exercise. Interestingly, quercetin supplementation further enhanced the activity of SOD induced by intense exercise.

To further evaluate the hepatoprotective effects of quercetin, ultrastructural examination was conducted by transmission electron microscopy (Figure 1(g)). Well-arranged rough endoplasmic reticula (RERs) with abundant attached ribosomes were observed in hepatocytes from the normal mice treated with or without quercetin. These structures were accompanied by many well-developed mitochondria with integral membranes and cristae. The nuclei of these hepatocytes were round with clear nuclear membranes, perinuclear cisterna, and nucleopores. Additionally, glycogenosomes were uniformly distributed. Intense exercise resulted in various degenerative changes. Fewer ribosomes were observed attached to swollen RERs (Figure 1(g) R); mitochondria appeared dilated or malformed (with missing cristae; Figure 1(g)  M); and the number of glycogenosomes was reduced. These intense exercise-induced ultrastructural abnormalities were somewhat reversed by quercetin; but the mitochondria remained swollen. The treatment of normal, nonoverexercised mice with quercetin had no effects on the serum aminotransferase levels, hepatic oxidative stress, or ultrastructural changes.

### 3.2. Quercetin Attenuated Intense Exercise-Induced Inflammatory Damage and ERS of Mouse Liver

**Figure 2:**
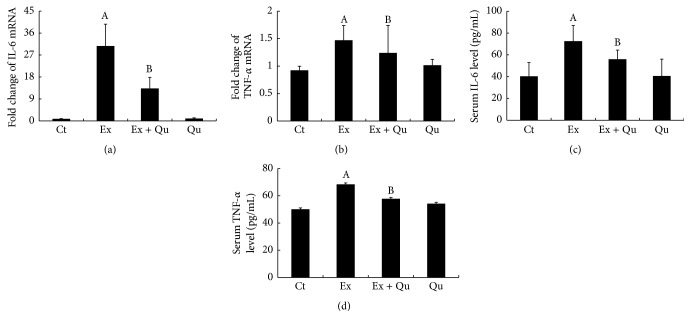
Influence of quercetin on inflammatory cytokines against intense exercise exposure in mouse liver. IL-6 (a) and TNF-*α* (b) mRNA expression levels; and IL-6 (c) and TNF-*α* (d) serum levels. The data are presented as the mean ± SD (*N* = 8 for ELISA, *N* = 6 for mRNA). Significant differences (*P* < 0.05) are indicated by different letters. A: versus Ct; B: versus Ex.

**Figure 3:**
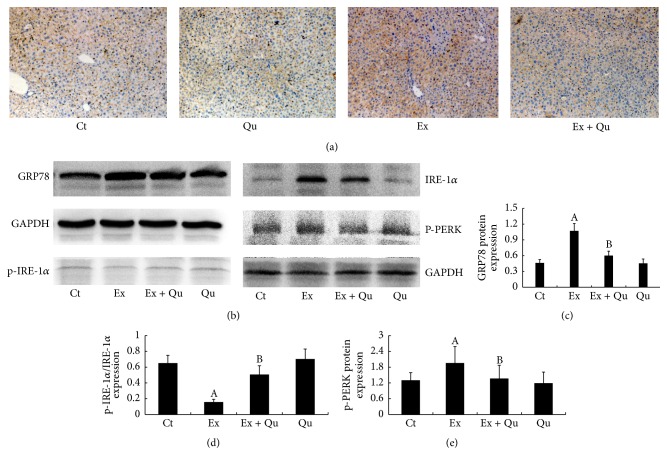
Influence of quercetin on ERS resulting from intense exercise exposure in mouse liver. The GRP78 (a, b, and c), p-IRE-1*α* (b and d), and p-PERK (b and e) protein expression levels were determined. The data are presented as mean ± SD (*N* = 3). Significant differences (*P* < 0.05) are indicated by the different letters. A: versus Ct; B: versus Ex.

Inflammatory stress was first evaluated in the present study. Markedly augmented hepatic IL-6 and TNF-*α* mRNA levels (Figures 2(a) and 2(b)) and increased serum inflammatory cytokine levels (Figures 2(c) and 2(d)) were observed in the intense exercise-challenged mice compared with the normal control mice. Subsequently, hepatic ERS, an initial cause of inflammation, was investigated. Hepatic GRP78, a classic ERS marker [28], was measured by Western blot and immunohistochemistry analyses. In correlation with inflammation, GRP78 protein expression was significantly upregulated following intense exercise (Figures 3(a), 3(b), and 3(c)), indicating that ERS potentially initiates inflammation in intense exercise-elicited liver injury. Furthermore, decreased expression of p-IRE-1*α* and increased level of p-PERK protein (Figures 3(b) and 3(d)), which are responsible for ERS-associated signaling [27], were observed in the intense exercise-exposed mice compared with control mice. The treatment of these intense exercise-exposed mice with quercetin had potent hepatoprotective effects via the inhibition of GRP78 and p-PERK, which increased the expression of p-IRE-1*α* and reduced the levels of inflammatory factors. These observations further supported the hypothesized link between ERS and inflammation. However, quercetin by itself had no effects on ERS or inflammatory damage compared with the normal, nontreated mice.

### 3.3. PI3K/Akt Pathway Potentially Mediated the Protective Effects of Quercetin against ERS

**Figure 4:**
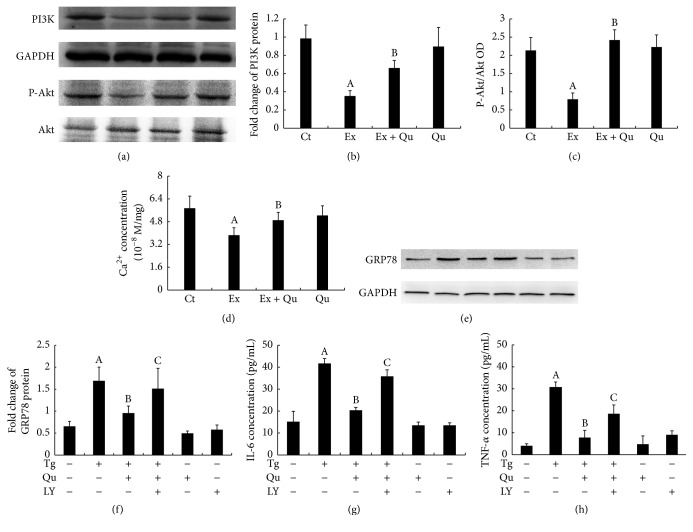
PI3K/Akt, Ca^2+^ dynamics, and ERS following intense exercise/Tg treatment and/or quercetin preconditioning. Hepatic PI3K (a and b) and Akt (a and c) protein levels, the Ca^2+^ concentration (d) in the ER, the GRP78 protein level (e and f) in mouse primary hepatocytes, and the amounts of IL-6 (g) and TNF-*α* (h) released from mouse primary hepatocytes into the culture medium were assessed. The data are presented as the mean ± SD (*N* = 10 for both the Ca^2+^ concentration determinations and ELISA, *N* = 3 for others). Significant differences (*P* < 0.05) are indicated by different letters. A: versus normal control (Ct); B: versus Tg; C: versus Qu + Tg: quercetin (100 *μ*M) plus Tg; Qu + Tg + LY: quercetin + Tg + LY294002 (30 nM).

The role of the PI3K/Akt pathway, which has been extensively associated with ERS [29] but has not been considered in overexercise models, in quercetin-induced hepatoprotection against ERS elicited by intense exercise remains unknown. As depicted in Figures 4(a), 4(b), and 4(c), the hepatic PI3K and p-Akt expression levels were significantly decreased with intense exercise and were accompanied by a decreased Ca^2+^ concentration in the ER compared with normal control mice (Figure 4(d)). The latter effect has been proposed to sequester GRP78 away from ERS transducers and accordingly promote ERS [30]. In contrast, quercetin supplementation effectively abolished the effects of intense exercise on PI3K/Akt expression and the corresponding Ca^2+^ dynamics.

To further elucidate the roles of the PI3K/Akt pathway in quercetin-mediated protection against ERS and inflammation-related damage, mouse primary hepatocytes were treated with quercetin, Tg (an activator of ERS via Ca^2+^ dynamic dysregulation), and LY294002 (an irreversible PI3K-specific inhibitor) following determination of the Tg concentration that stimulated ERS (in a dose-dependent manner, i.e., 0, 0.1, 0.5, and 1.0 *μ*M; data not shown). Similar results for GRP78 expression and inflammatory stress induced by Tg treatment were observed in the intense exercise-exposed mice. As expected, the quercetin intervention effectively reduced ERS and inflammation compared with the Tg-stimulated hepatocytes. Nevertheless, the protective effects of quercetin on ERS and inflammatory injury were partially abolished by preincubation of mouse primary hepatocytes with a PI3K inhibitor, LY294002. Taken together, these results indicate that Ca^2+^ mediates the preventive effects of quercetin on ERS via the PI3K/Akt pathway and alleviates inflammation.

### 3.4. Potential Signaling Pathway Associated with ERS-Mediated Inflammatory Damage in Quercetin-Mediated Prophylaxis against Intense Exercise-Stimulated Hepatotoxicity

**Figure 5:**
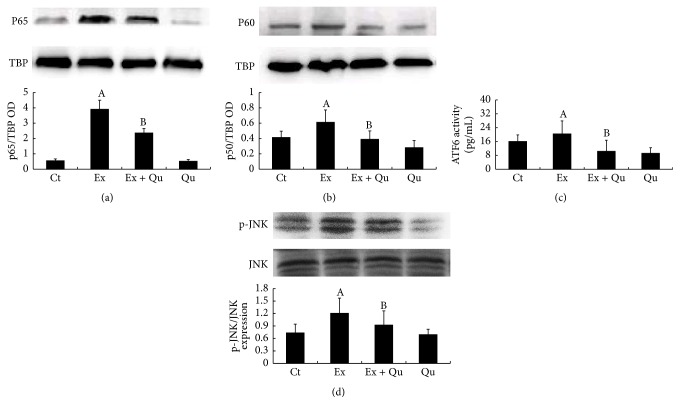
Protein expression of NF-*κ*B (p65 and p50 subunits) and JNK and activity level of ATF6 in mouse liver following intense exercise exposure with or without quercetin treatment. p65 (a) and p50 (b) subunit protein expression, ATF6 activity (c), and JNK protein expression (d) in nuclear fractions were determined. Three independent mouse liver isolations were performed for the protein measurements, in addition to the eight for ELISA. Significant differences (*P* < 0.05) are indicated by the different letters. A: versus Ct; B: versus Ex.

To investigate the signal transduction mechanisms, we examined the effects of the well-studied cross-talks among three molecules, that is, NF-*κ*B, JNK, and ATF6, on the quercetin-induced protection against ERS-triggered inflammatory damage. The core components of the classical activation pathway of NF-*κ*B, p65, and p50 were determined by Western blot analyses of nuclear and cytoplasmic extracts. As illustrated in Figures 5(a) and 5(b), the nuclear translocation of p65 and p50 was evidently promoted by intense exercise stimulation compared with that in the normal control mice. Similarly, the NF-*κ*B, ATF6, and p-JNK levels were also significantly increased by intense exercise exposure (Figures 5(c) and 5(d)). Compared with the levels of JNK and ATF6, that of NF-*κ*B exhibited a greater increase as a result of continuous intense exercise. Impressively, we observed marked decreases in the NF-*κ*B, ATF6, and p-JNK levels in the quercetin-treated mice following intense exercise, accompanying the reduction in inflammatory cytokines. These results imply that the quercetin-induced alleviation of ERS and inflammatory injury may be correlated with intense exercise-evoked signal transduction, especially NF-*κ*B nuclear translocation.

**Figure 6:**
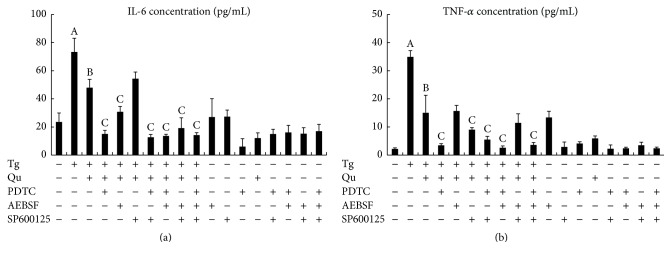
Effects of quercetin and Tg on the release of IL-6 (a) and TNF-*α* (b) from mouse primary hepatocytes and the corresponding signaling pathways involved in ERS-mediated inflammation. The data are expressed as the mean ± SD from at least six independent hepatocyte isolations performed in triplicate. Significant differences (*P* < 0.05) are indicated by the different letters. A: versus normal control (Ct); B: versus Tg; C: versus Qu + Tg.

To further characterize the roles of the above signal transduction mechanisms, mouse primary hepatocytes were preincubated with PDTC (an NF-*κ*B inhibitor, 300 *μ*M), SP600125 (a JNK inhibitor, 20 *μ*M), and AEBSF (an ATF6 inhibitor, 300 *μ*M) for 2 hours before Tg treatment and quercetin intervention. As shown in Figure 6, PDTC and SP600125 separately or synergistically inhibited the release of TNF-*α* from hepatocytes coincubated with Tg and quercetin. However, SP600125 combined with Tg and quercetin treatment had no dramatic effects on IL-6 release in hepatocytes. Furthermore, PDTC exposure resulted in a significant decrease in IL-6 to a level similar to that in untreated hepatocytes. Remarkably, PDTC had a stronger inhibitory effect on quercetin-mediated protection against ERS-induced inflammatory damage than SP600125. Furthermore, cotreatment with PDTC and AEBSF led to reduced levels of inflammatory cytokines (TNF-*α* and IL-6) in Tg- and quercetin-incubated hepatocytes but higher levels than those in normal controls. Treatment with AEBSF alone had no significant influence on the TNF-*α* level in Tg-treated hepatocytes. Notably, dramatically reduced inflammatory cytokine levels were observed following treatment of the Tg- and quercetin-exposed hepatocytes with the three inhibitors together. Treatment with each inhibitor alone had no effect on intact mouse hepatocytes. Furthermore, the inhibitors did not exhibit any cytotoxicity.

Collectively, these findings indicate that the PI3K/NF-*κ*B pathway plays a crucial role in the protective effects of quercetin against intense exercise/ERS-triggered inflammatory damage in hepatocytes.

## 4. Discussion

In the present study, intense exercise significantly induced liver damage, resulting in aminotransferase release, oxidative stress, and ultrastructural abnormalities, in agreement with the results of previous studies [4, 6, 7, 31]. Accumulating evidence has revealed that inflammation, a well-described series of events initiated by tissue injury, plays major deleterious roles in several conditions (e.g., sepsis and rheumatoid arthritis). Furthermore, inflammation is especially associated with the pathogenesis of liver diseases [32]. The observed increases in the IL-6 and TNF-*α* levels, in accordance with other* in vivo* observations [2, 4, 33], further support the crucial role of inflammatory stress in intense exercise. In current study, quercetin treatment (100 mg/kg·bw for 5 weeks) effectively attenuated inflammatory stress during intense exercise stimulation and Tg exposure both* in vivo* and* in vitro*. Most animal studies support the protection against exercise-induced oxidative stress and inflammation. Lin et al. [20] have found that quercetin-3-O-gentiobiose possessed its anti-inflammatory activity via reducing the levels of TNF-*α* and IL-6 in exhaustive swimming rats in a dose-independent manner. Our earlier research has revealed that quercetin protects the mouse liver from inflammatory stress resulting from toxic insults [25]. Similarly, quercetin has been reported to possess potential hepatoprotective effects against high-fat diets [34] and nickel-induced conditions by counteracting inflammatory damage [35], whereas, in another study from HFD-fed mice, dietary quercetin dehydrate at dose of 10 mg/kg·bw/day for 8 weeks did not ameliorate strenuous exercise-induced inflammation in skeletal muscle [36]. We supposed that animal models, supplement form and quercetin dosage partly contributed to the inconsistency. Unfortunately, only a few human trials observed quercetin-induced changes in inflammation or plasma antioxidant status. Phillips et al. [37] have found that a mix of antioxidants containing quercetin (tocopherols, docosahexaenoate, and hesperetin) attenuates IL-6 release after eccentric exercise in untrained males, demonstrating the cooperative effects of quercetin and other antioxidants on inflammation, whereas Konrad et al. [38] have concluded that acute supplementation of 1000 mg quercetin plus 400 mg isoquercetin before heavy exertion among endurance athletes did not prevent postexercise inflammation, although it caused a strong increase in plasma quercetin levels. O'Fallon et al. [34] have shown that quercetin aglycone supplementation (1,000 mg/d, for 7 d before and 5 d after exercise) had no effect on markers of eccentric exercise-induced muscle damage or inflammation among healthy subjects, paralleling with no change in plasma IL-6 levels before and after exercise with/without quercetin supplementation. Notably, quercetin could improve exercise performance. Recently, Askari et al. [39] demonstrated that 500 mg quercetin plus 250 mg vitamin C daily for 8 weeks revealed positive effects on endurance performance among male students with athletic history. The negative effects of quercetin obtained from above human studies during exercise might be due to diverse exercise manner and durations, the dose and composition of dietary supplement, and subject populations, for example, healthy and untrained, or highly trained athletes who are known to possess increased antioxidant defenses. These incompatible findings from animal and human models have still to be fully elucidated. Notably, the metabolic transformation and accumulation of quercetin between animal and human could also partly explain its bioavailability variations. In human, quercetin is metabolized in small intestines, colon, liver, and kidney and is conjugated to methyl and sulfate groups and glucuronic acid to generate its major conjugates. Moreover, the specific accumulation of quercetin conjugates and corresponding bioactivity in liver and/or muscle is still unclear. In all, our present experimental data suggest that quercetin could be a promising pharmacological agent used to prevent intense exercise-related liver damage due to its naturally occurring antioxidant and anti-inflammatory activities. However, further studies in human trials are still needed to be conducted to reach a clear conclusion.

In addition to its ability to decrease the production of proinflammatory cytokines, the regulation of the endogenous anti-inflammatory system by quercetin has attracted attention. Among several potential mechanisms, ERS is considered to be an initial determining factor for stress-associated pathophysiologic disorders. ERS has been well-demonstrated to play an extremely important role in the pathogenesis of inflammation-induced liver injury [16, 28]. Generally, GRP78 binds to transducers (IRE-1*α* and PERK), which are maintained in inactive states under physiological conditions. However, our results showed that intense exercise-exposed mice exhibited marked ERS manifested by increased GRP78 expression and the subsequent activation of PERK and IRE-1*α*. The dysregulation Ca^2+^ dynamics via PI3K/Akt has been suggested to greatly contribute to the sequestration of GRP78 away from ERS transducers. This activity accordingly evokes ERS in apoptosis-induced tumor cells [29], reoxygenated hypoxic neonatal rat cardiomyocytes [30], and intestinal epithelial cells incubated with an ERS inducer [40]. Additionally, several studies have demonstrated that the activation of PI3K promotes hepatoprotective effects from posthepatectomy, ischemic preconditioning, or cyclic AMP [41–43]. These data, to some extent, suggest that PI3K suppression may contribute to the ERS-induced inflammatory damage of the mouse liver following intense exercise in association with a Ca^2+^ disturbance.

Plant-derived flavonoids may act as nonstressful and noncytotoxic PI3K inducers and accordingly minimize cellular stress. Quercetin accumulates in liver tissue following metabolism and accounts for 60%–75% of dietary flavonoids [44]. Importantly, it has been reported to protect hepatocytes against oxidative stress via PI3K activation [45]. In the current study, quercetin induced PI3K activation and Akt phosphorylation and maintained a Ca^2+^ level in the ER, thereby reducing intense exercise-derived ERS. Furthermore, the specific PI3K inhibitor LY294002 abolished the protective effects of quercetin on GRP78 and inflammatory cytokines in Tg-treated hepatocytes. The inhibitory effect of LY294002 is well-known to be based on its ability to block the ER Ca^2+^ ATPase SERCA, which is responsible for transporting Ca^2+^ from the cytosol to the ER [46]. PI3K activation has been suggested to lead to the recovery of SERCA and consequently to Ca^2+^ homeostasis [47]. Accordingly, these findings imply that regulation of the PI3K/Akt signaling pathway contributes, to some extent, to the protective effects of quercetin against ERS. Conversely, opposite effects of quercetin on PI3K in LS180 cells and Caco-2 cells have been reported by another group, indicating a dual role of quercetin in PI3K activation [40]. Differing doses and durations of exposure to ERS inducers likely contributed to these reported inconsistencies.

The present study also explored the potential mechanisms of ERS-mediated inflammation in quercetin hepatoprotection. A number of studies [13, 35] have demonstrated that NF-*κ*B, JNK, and ATF6 are critical for transducing ERS signals to initiate inflammatory responses. The activity of NF-*κ*B, as demonstrated by p65 and p50 nuclear translocation, exhibited a greater increase compared with the activities of JNK and ATF6. Furthermore, the release of IL-6 from hepatocytes coincubated with quercetin and Tg was significantly reduced by AEBSF. Similarly, SP600125 decreased the release of TNF-*α* in quercetin- and Tg-treated hepatocytes. AEBSF had no significant effect on TNF-*α*, and SP600125 did not further block IL-6 release from hepatocytes following quercetin and Tg treatment. Both inflammatory cytokines were significantly blocked by the NF-*κ*B inhibitors, applied singly or in combination, indicating that NF-*κ*B is primarily responsible for quercetin-mediated hepatoprotection against ERS-induced inflammation. The involvement of NF-*κ*B in ERS-activated inflammation has also been established by other studies of the effects of 4-phenylbutyrate on lipopolysaccharide-induced lung damage [48], cardiomyocyte hypoxia/reoxygenation [49], and IL-1*β* production [50]. Further, quercetin has been found to inhibit NF-*κ*B-mediated HepG2 cell damage [51]. Such protective effects, which occur via NF-*κ*B sequestration, have also been observed for other flavonoids (e.g., morin and carnosol) [52, 53]. These* in vivo* and* in vitro* data show that quercetin may suppress the expression of IL-6 and TNF-*α* by blocking the recruitment of NF-*κ*B to respective gene promoters upon ERS exposure.

## 5. Conclusions

The results of this study demonstrate the beneficial effects of quercetin on liver damage following exhaustive exercise. Quercetin supplementation may be an effective strategy to offset increases in oxidative stress, inflammation, and ERS, which have been linked with stressful exercise. These results may lead to the development of nutritional regimens that decrease the risk of liver injury, which can be an issue for military personnel and athletes engaged in cycling, triathlons, cross-country skiing, rowing, and distance running. Furthermore, our findings highlight PI3K and NF-*κ*B as potential candidate targets for the prevention of tissue damage induced by intense exercise. Despite these findings, the molecular mechanisms and interactions of ERS and inflammatory damage remain unknown.

## Supplementary Material

Supplementary Fig 1. Weight change. The data are expressed as the mean ± SD (*N* = 10). a: *P* < 0.05 vs. normal control (Ct); Ex: intense exercise; Ex + Qu: intense exercise plus quercetin (100 mg/kg·bw); Qu: quercetin.
