# Supplementary figures and images for: Hepatoprotective Effect of Quercetin on Endoplasmic Reticulum Stress and Inflammation after Intense Exercise in Mice through Phosphoinositide 3-Kinase and Nuclear Factor-Kappa B
Source: Oxid Med Cell Longev. 2016 Jul 18;2016:8696587. doi: 10.1155/2016/8696587 (PMC4967689; doi:10.1155/2016/8696587)

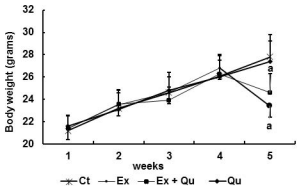

Supplementary Fig 1

Supplement: Supplementary file 1 — Supplementary Fig 1. Weight change. The data are expressed as the mean ± SD (N = 10). a: P < 0.05 vs. normal control (Ct); Ex: intense exercise; Ex + Qu: intense exercise plus quercetin (100 mg/kg·bw); Qu: quercetin. [file 8696587.f1.pdf]
